# Is mesh essential in laparoscopic hiatal hernia repair? analysis of 30-day outcomes from the ACS-NSQIP database over eight years

**DOI:** 10.3389/fsurg.2025.1607633

**Published:** 2025-06-13

**Authors:** Clayton Wyland, Sonia Parievsky, Rami Madani, David M. Richter, Alain Elian, Saad Shebrain

**Affiliations:** Department of Surgical Sciences, Division of General Surgery, Western Michigan University Homer Stryker M.D. School of Medicine, Kalamazoo, MI, United States

**Keywords:** hiatal hernia, paraesophageal hernia, antireflux surgery, hiatal hernia repair, fundoplication, laparoscopic, mesh

## Abstract

**Introduction:**

Use of mesh to reinforce laparoscopic hiatal hernia repair (LHHR) has been a popular topic of debate among foregut surgeons in recent years. Augmentation with mesh appears to reduce short-term recurrence rates; however, little is known about other important short-term outcomes. Such information is critical to delineating the optimal treatment approach for hiatal hernia. Therefore, this study evaluated various 30-day outcomes in patients who underwent LHHR, both with and without mesh.

**Methods:**

American College of Surgeons National Surgical Quality Improvement Program (ACS-NSQIP) database was used to identify patients who underwent LHHR from 2010–2017. Patients were sorted into one of two cohorts: LHHR with mesh or LHHR without mesh. 30-day postoperative mortality, morbidity, length of hospital stay (LOS), operative time, reoperation, and readmission rates were analyzed using SPSS.

**Results:**

A total of 24,488 patients underwent LHHR—9,710 (37.4%) with mesh and 15,318 (62.6%) without mesh. Both groups had similar demographic characteristics. At 30-days, there were no differences between the groups regarding mortality (0.6% vs. 0.6%, *p* = .990), serious morbidity (3.8% vs. 3.5%, *p* = .135), overall morbidity (6.4% vs. 6.2%, *p* = .468), and return to the operating room (2.6% vs. 2.6%, *p* = .945). However, patients in the mesh group had an increased readmission rate (6.6% vs. 5.8%, *p* = .013), median [IQR] operative time (147 [108,197] vs. 130 [91,175] minutes, *p* < .001), and mean LOS (2.9 vs. 2.7 days, *p* = .002).

**Conclusion:**

In this large retrospective cohort study, LHHR with mesh was associated with increased operative time, LOS, and hospital readmission. However, there were no differences in mortality or overall morbidity. These findings provide much needed context to consider prior to employing mesh in LHHR.

## 1 Introduction

Hiatal hernias (HH) are characterized by protrusion of abdominal contents, most commonly the stomach, through the esophageal hiatus and into the mediastinum. The resulting anatomical disruption of the gastroesophageal junction (GEJ) reduces lower esophageal sphincter pressure, increases distensibility, and impairs esophageal peristalsis (1, 2). Together, these physiological derangements result in increased reflux of gastric contents, and symptoms of gastroesophageal reflux disease (GERD) (3). The most comprehensive classification system, established nearly a century ago, describes four types of HH (4). Type I (“sliding”) HH, occurs when the GEJ and some of the gastric cardia “slide” through the esophageal hiatus. Type I hernias account for 95% of all HH. In type II (paraesophageal) HH, the GEJ remains in place, while a portion of the gastric fundus herniates into the mediastinum, alongside the esophagus. Type III (mixed) HH involves migration of both the GEJ and a portion of the fundus into the mediastinum; this frequently involves both sliding and paraoesophageal components (5). In type IV (giant paraesophageal) HH, both the stomach and proximal abdominal organs—such as the colon or spleen—herniate into the chest (6). Importantly, only types II–IV involve fixed anatomical defects and, therefore, are considered “true” hernias (5, 7).

The size of the HH correlates with the degree of GEJ dysfunction and, therefore, the symptoms a patient experiences (8, 9). While small HH are often asymptomatic and do not require intervention, large and symptomatic HH often require surgery. Although data pertaining to the United States is limited, international studies have found the laparoscopic approach is employed for nearly all repairs (10). LHHR involves several key steps, including reduction of the hernia contents, takedown of the short gastric vessels, dissection of the hiatal hernia sac, excision of hernia sac, and mobilization of the esophagus. This is followed by primary closure of the diaphragmatic hiatus by approximating the left and right crurae of the diaphragm—a procedure known as cruroplasty. The procedure is typically completed with fundoplication to help prevent future reflux (11). Additionally, many surgeons believe combining mesh with suture-based cruroplasty can offer a more durable repair. This involves placing mesh over the crural repair site and securing it in place with sutures or tacks. Several types of mesh can be utilized, including absorbable and non-absorbable (biologic and synthetic) mesh (12).

However, the use of mesh in reinforcing the repair has remained a popular subject of debate in foregut surgery over recent years (13). Several studies have assessed recurrence rates as the primary outcome of hiatal hernia repairs using mesh, revealing promising reductions in short-term recurrence rates with mesh augmentation. However, a plethora of long-term studies have failed to demonstrate a sustained reduction in recurrence rates when patients are monitored over multiple years (14, 15). The inconsistency in the long-term efficacy of treatment has prompted further discussions about best practice. The ongoing debate warrants the necessity for further research to delineate the most optimal treatment approach, potentially by investigating disparities in patient outcomes between the two repair methods.

Therefore, this study evaluated various 30-day postoperative outcomes­ in patients who underwent LHHR, both with and without mesh. Primary outcomes included morbidity and mortality; secondary outcomes included length of hospital stay (LOS), operative time, and rates of reoperation and hospital readmission.

## 2 Methods

### 2.1 Study population

The American College of Surgeons National Surgical Quality Improvement Program (ACS-NSQIP) database was queried to identify patients ≥18 years of age who underwent LHHR between the years of 2010 and 2017. Current Procedural Terminology (CPT) codes were used to separate eligible patients into two cohorts: LHHR with mesh (CPT 43282: “Laparoscopy, surgical, repair of paraesophageal hernia, includes fundoplasty, when performed; with implantation of mesh”) and LHHR without mesh (CPT 43281: “Laparoscopy, surgical, repair of paraesophageal hernia, includes fundoplasty, when performed; without implantation of mesh”). For the purposes of this study, the terms “hiatal hernia” and “paraesophageal hernia” were used interchangeably; a patient with any of the International Classification of Disease 9th and 10th edition (ICD-9 and -10) codes listed in Table 1 was considered to have a hiatal hernia. All study protocols and procedures were reviewed by the Institutional Review Board at Western Michigan University Homer Stryker MD School of Medicine.

**Table 1:** Hiatal hernia diagnoses based on ICD-9 and -10 codes.

Diagnosis	ICD-9/-10 Code(s)
Diaphragmatic hernia	K44
Diaphragmatic hernia with gangrene	551.3, K44.1
Diaphragmatic hernia with obstruction	552.3
Diaphragmatic hernia with obstruction, without gangrene	K44.0
Diaphragmatic hernia without obstruction or gangrene	553.3, K44.9

### 2.2 Study outcomes

Primary study outcomes included rates of morbidity (minor, serious, and overall) and mortality at 30-days post-procedure. For the purposes of this study, minor morbidity was defined as documentation of ≥1 minor ACS-NSQIP complications (pneumonia, superficial or deep incisional surgical site infection [SSI], unplanned intubation, urinary tract infection, or deep vein thrombosis. Serious morbidity was defined as documentation of ≥1 major ACS-NSQIP complication(s) (organ space SSI, wound disruption, cerebrovascular accident, cardiac arrest requiring CPR, myocardial infarction, pulmonary embolism, mechanical ventilation for ≥48 h, acute renal failure, progressive renal insufficiency, transfusion of >4 units packed red blood cells, sepsis, and septic shock). Overall morbidity was defined as documentation of ≥1 source of minor or serious morbidity. These classifications are consistent with those employed by previous studies involving the ACS-NSQIP database (16). Secondary outcomes included mean total LOS, operative time, and rates of reoperation and hospital readmission.

### 2.3 Statistical analysis

Descriptive statistics were used to summarize patient demographics, preoperative risk factors, and postoperative outcomes. Continuous variables were analyzed using Student's *t*-test or the Mann–Whitney *U* test and presented as mean (SD) or median (IQR) as appropriate. Categorical variables are presented by frequency (*n*) and percent (%) and analyzed using the Chi-Squared or Fisher Exact tests as appropriate. Statistical significance was set at *p* < 0.05. Statistical analyses were performed using SPSS software (IBM SPSS Statistics for Windows, Version 27.0, IBM Corp.).

## 3 Results

### 3.1 Patients

A total of 24,488 patients were identified, of which 9,170 (37.4%) underwent LHHR with mesh and 15,318 (62.6%) underwent LHHR without mesh. 17,596 (72%) of the identified patients were female. Demographic characteristics (sex and race) were similar between the groups. However, patients who underwent LHHR with mesh were more likely to have hypertension (52.4% vs. 48.8%, *p* < .001) and dyspnea (13.6% vs. 11.7%, *p* < .001). Conversely, patients who had LHHR without mesh were younger (60.2 years vs. 63.2 years old, *p* < .001) and more likely to have diabetes (10.0% vs. 8.5%, *p* < .001). Notably, patients over the age of 65 were significantly more likely to have repair with mesh (47% vs. 39%, *p* < .001). Information regarding additional demographics and comorbidities can be found in Table 2.

**Table 2:** Demographic and perioperative characteristics of patients who underwent laparoscopic hiatal hernia repair with and without mesh.

Demographic characteristics and preoperative data	Repair with Mesh (*N* = 9,170)	Repair without Mesh (*N* = 15,318)	Total (*N* = 24,488)	*p*-value
Demographic Characteristics				
Sex, female, *n* (%)	6,573 (71.7)	11,023 (72.0)	17,596 (71.9)	.118
Age, mean (SD)	63.21 (13.7)	60.20 (14.8)	61.32 (14.5)	<.001
Age Class				<.001
≤65 years	4,800 (52.3)	9,217 (60.2)	14,017 (57.2)	
>65 years	4,370 (47.7)	6,101 (39.8)	10,471 (42.8)	
Race, *n* (%)				<.001
White	8,190 (89.3)	13,149 (85.8)	21,339 (87.1)	
Black or African American	389 (4.2)	954 (6.2)	1,343 (5.5)	
BMI, *n* (%)				<.0001
Underweight (BMI < 18.5)	58 (0.6)	112 (0.7)	170 (0.7)	
Normal (BMI: 18.5–24.9)	1,551 (17.0)	2,322 (15.3)	3,828 (15.9)	
Overweight (BMI: 25.0–29.9)	3,376 (37.0)	4,952 (32.5)	8,328 (34.2)	
Obese: Class I (BMI 30.0–34.9)	2,550 (28.0)	4,133 (27.2)	6,683 (27.5)	
Obese: Class II (BMI 35.0–39.9)	1,091 (12.0)	2,052 (13.5)	3,143 (12.9)	
Obese: Class III (BMI >40)	487 (5.3)	1,649 (10.8)	2,136 (8.8)	
Surgery acuity, *n* (%)				
Elective	7,773 (91.1)	13,334 (91.7)	21,107 (91.5)	.087
Emergency	176 (1.9)	311 (2.0)	487 (2.0)	.547
Medical Comorbidities, *n* (%)				
Diabetes	781 (8.5)	1,536 (10.0)	2,317 (9.5)	<.001
Smoking	799 (8.7)	1,425 (9.3)	2,224 (9.1)	.120
Dyspnea at rest/moderate exertion	1,259 (13.7)	1,796 (11.7)	3,055 (12.5)	<.001
Functional Status^a^	159 (1.7)	277 (1.8)	436 (1.8)	.654
Ventilator-dependent	10 (0.1)	10 (0.1)	20 (0.1)	.246
COPD	537 (5.9)	749 (4.9)	1,286 (5.3)	.001
Congestive heart failure	37 (0.4)	64 (0.4)	101 (0.4)	.866
Hypertension requiring medications	4,803 (52.4)	7,474 (48.8)	12,277 (50.1)	<.001
Dialysis-dependent	19 (0.2)	18 (0.2)	37 (0.2)	.080
Disseminated Cancer	14 (0.2)	27 (0.2)	41 (0.2)	.662
Chronic wound	21 (0.2)	61 (0.4)	82 (0.3)	.027
Steroid use for chronic conditions^b^	382 (4.2)	553 (3.6)	935 (3.8)	.028
Weight loss^c^	156 (1.7)	315 (2.1)	471 (1.9)	.050
Bleeding Disorders^d^	183 (2.0)	303 (2.0)	486 (2.0)	.923
Transfusion prior to surgery^e^	42 (0.5)	51 (0.3)	93 (0.4)	.123
Presentation with sepsis				.437
None	8,992 (98.5)	15,070 (98.5)	24,062 (98.5)	
SIRS	129 (1.4)	199 (1.30)	328 (1.30)	
Sepsis	7 (0.1)	22 (0.1)	29 (0.1)	
Septic Shock	2 (0.0)	3 (0.0)	5 (0.0)	
ASA Class^f^				.041
ASA-1	107 (1.2)^a^	248 (1.6)^b^	355 (1.4)	
ASA-2	4,477 (48.8)^a^	7,352 (48.0)^a^	11,829 (48.30	
ASA-3	4,354 (47.5)^a^	7,349 (48.0)^a^	11,703 (47.8)	
ASA-4	227 (2.5)^a^	354 (2.3)^a^	581 (2.4)	

SD, standard deviation; BMI, body mass index; COPD, chronic obstructive pulmonary disease; SIRS, systemic inflammatory response syndrome; ASA, American society of anesthesiologists.

^a^ Defined as being totally or partially dependent at presentation.

^b^ Defined as >30 consecutive days of oral corticosteroids.

^c^ Defined as >10% loss of body weight within the six months preceding surgery.

^d^ Defined as ICD-10 codes D65–69: coagulation defects, purpura, and other hemorrhagic conditions (e.g., vitamin K deficiency, hemophilia, thrombocytopenia, chronic anticoagulation therapy that was not discontinued prior to surgery).

^e^ Defined as requiring ≥1 unit(s) of packed red blood cells within the 72 h preceding surgery.

^f^ Each subscript letter denotes a subset of CPT categories whose column proportions are not significantly different.

### 3.2 30-Day postoperative outcomes

There were no significant differences between the groups in mortality (0.6% vs. 0.6%, *p* = .990), minor morbidity (4.3% vs. 4.1%, *p* = .561), serious morbidity (3.8% vs. 3.5%, *p* = .135), or overall morbidity (6.4% vs. 6.2%, *p* = .468). Mesh use was associated with significantly more myocardial infarction (0.2% vs. 0.4%, *p* = .006), albeit the total incidence was quite low. Reoperation rates did not differ between the groups (2.6% vs. 2.6%, *p* = .945). Repair with mesh was associated with increased readmission (6.6% vs. 5.8%, *p* = .013), increased median (IQR) operative time [147 (108, 197) vs. 130 (91,175) minutes, *p* < .001], and increased LOS (3.0 vs. 2.9 days, *p* = .002). The complete analysis can be found in Table 3.

**Table 3:** 30-day postoperative outcomes.

Postoperative outcomes	Repair with mesh (*N* = 9,170)	Repair without mesh (*N* = 15,318)	Total (*N* = 24,488)	*p*-value
30-day outcomes, *n* (%)				
Mortality	51 (0.6)	85 (0.6)	136 (0.6)	.990
Serious Morbidity	351 (3.8)	530 (3.5)	881 (3.6)	.135
Minor Morbidity	390 (4.3)	628 (4.1)	1,018 (4.2)	.561
Overall Morbidity	587 (6.4)	945 (6.2)	1,532 (6.3)	.468
Reoperation/Return to OR	239 (2.6)	397 (2.6)	636 (2.6)	.945
Readmission	557 (6.6)	829 (5.8)	1,386 (6.1)	.013
Operative time and LOS				
Operative time (minutes), mean (SD)	160 (75)	136 (70)	145 (73)	<.0001
Operative time, median (IQR)	145 (106, 195)	123 (87, 170)	131 (94, 180)	<.001
LOS, median (IQR)	2 (1, 3)	2 (1, 3)	2 (1, 3)	.002
Postoperative complications, *n* (%)				
Superficial incisional SSI	38 (0.4)	82 (0.5)	120 (0.5)	.190
Deep incisional SSI	8 (0.1)	12 (0.1)	20 (0.1)	.812
Organ/Space SSI	64 (0.7)	109 (0.7)	173 (0.7)	.902
Wound dehiscence	2 (0.0)	8 (0.1)	10 (0.0)	.254
Pneumonia	171 (1.9)	259 (1.7)	430 (1.80	.316
Unplanned reintubation	94 (1.0)	135 (0.9)	229 (0.9)	.258
Pulmonary embolism	52 (0.6)	69 (0.5)	121 (0.5)	.208
Failure to wean of ventilation (>48 h)	77 (.08)	115 (0.8)	192 (0.8)	.445
Progressive renal insufficiency	11 (0.1)	24 (0.2)	35 (0.1)	.462
Acute renal failure	15 (0.20	20 (0.1)	35 (0.1)	.508
Urinary Tract Infection	98 (1.1)	178 (1.2)	276 (1.1)	.503
Stroke/cerebrovascular accident	11 (0.1)	13 (0.1)	24 (0.1)	.396
Cardiac arrest requiring CPR	23 (0.3)	29 (0.2)	52 (0.2)	.312
Myocardial infarction	40 (0.4)	36 (0.2)	76 (0.3)	.006
Bleeding requiring transfusion	114 (1.20	182 (1.2)	296 (1.2)	.703
Deep vein thrombosis	42 (.50	63 (0.4)	105 (0.4)	.588
Sepsis	54 (0.6)	103 (0.7)	157 90.6)	.428
Septic Shock	53 (0.6)	83 (0.5)	136 (0.6)	.713
Discharge destination, *n* (%)				<.001
Home	8,169 (89.1)^a^	13,993 (91.4)^b^	22,162 (90.5)	
Skilled Care Nursing	303 (3.3)^a^	468 (3.1)^a^	771 (3.1)	
Acute Care	25 (0.3)^a^	33 (0.2)^a^	58 (0.2)	

SSI, surgical site infection; CPR, cardiopulmonary resuscitation.

^a^ Denotes a subset of discharge locations whose column proportions are not significantly different from those with the same superscript letter.

^b^ Denotes a subset of discharge locations whose column proportions are not significantly different from those with the same superscript letter.

## 4 Discussion

In this retrospective cohort study comparing LHHR performed with and without mesh, no significant differences were observed in 30-day mortality, minor morbidity, serious morbidity, or overall morbidity. Importantly, mesh use was associated with higher rates of hospital readmission and longer operative time, as compared to suture-only repair. Mesh use was also associated with increased LOS; however, the difference in LOS is likely not clinically significant, given its small magnitude. Patients in the mesh cohort experienced more myocardial infarction (MI), although the overall incidence was quite low, and this is likely not clinically significant. However, it is possible that the increased incidence of MI was impacted by patient factors, as those who underwent repair with mesh were more likely to have hypertension and be of older age. To our knowledge, this study represents the largest and most comprehensive analysis of 30-day postoperative outcomes to date. Although a previous study similarly found no significant differences in 30-day mortality or overall morbidity (17), this is the first study to establish an association between LHHR with mesh, prolonged operative time, and increased readmission rates.

However, it must be noted that the increased rate of readmission observed among those who underwent LHHR with mesh may be related to patient age and hernia size. In our study, patients >65 years of age were more likely to undergo LHHR with mesh. Although we cannot definitively say why this was the case, it was likely related to hernia size—evidence suggests that hernia size may increase with age (18–20). As hernia size appears to be associated with increased readmission (21, 22), it is possible that the increased readmission rates seen in our study may be a function of hernia size and, therefore, patient age.

### 4.1 Clinical applications

In our experience, the choice to employ mesh during LHHR is driven largely by surgeon preference—some surgeons routinely use mesh, others reserve it for specific patient groups, while some avoid it altogether. Due to this variability in practice, several studies have examined rates of recurrence for mesh vs. suture-only repair, with some stratifying based on defect size. In the short-term, it appears that mesh does not significantly impact recurrence rates, regardless of size (23, 24). However, in the longer-term, the evidence is less clear—some studies report lower recurrence rates when mesh is employed, while others have found no significant differences (25). It must be noted that many patients display radiological evidence of recurrence but remain asymptomatic, raising questions regarding the clinical utility of such findings (26). Patient-centered outcomes, mainly quality of life, are another factor to consider. Mesh use appears to be associated with less reflux symptoms in the year following surgery, but many patients report worsening dysphagia postoperatively (27). Interestingly, patients who undergo repair with biological mesh tend to score higher on short-term quality of indices, despite higher rates of dysphagia (28).

Given conflicting evidence regarding the impact of mesh on HH recurrence and quality of life, the potential for mesh-related complications must be considered. Therefore, our results may offer valuable context to inform clinical decision-making. Importantly, our study suggests that mesh placement offers a comparable safety profile relative to suture cruroplasty. However, we observed a notable difference in operative time, with mesh placement accounting for an additional 24 min in the operating room. Although this may appear to be somewhat inconsequential, the additional minutes can result in sizable direct and indirect costs for the payer and may also contribute to strain on healthcare resources (29, 30). Such costs accrue in addition to the substantial costs of the mesh deployed during the operation (31). Moreover, mesh placement was associated with increased hospital readmission—an event that correlates with poorer health outcomes, has negative impacts on quality of life, and may strain healthcare resources (32, 33). As studies evaluating the efficacy of mesh repair remain equivocal, and mesh use should be used at the discretion of the surgeon (14), our findings provide important context to consider.

### 4.2 Limitations

Our study has some limitations. First, retrospective studies come with inherent limitations. One such limitation is potential confounding. For this reason, we assessed a multitude of comorbidities to identify possible confounds. Although information necessary to reach a definitive conclusion is not available in the ACS-NSQIP database, differing rates of COPD and hypertension may have contributed to higher rates of readmission in the mesh-reinforced group. Additionally, analysis of large existing databases comes with a risk of selection bias and coding errors. However, the significant diversity of hospitals included, and the intensive methodology of data collection utilized by the ACS-NSQIP database helps mitigate these risks. Second, the ACS-NSQIP database only tracks 30-day outcomes; therefore, longer-term sources of morbidity or mortality could be missed. Additionally, despite having more than 270 variables in the ACS-NSQIP database, these variables are global (i.e., applied to many surgical specialties and are not procedure or diagnosis-specific); therefore, information regarding the size of HH, whether primary or recurrent, and the specific mesh or reinforcement material used were not available for analysis. Over the eight years included in this study, the reason for readmission was not consistently recorded for all years in the ACS-NSQIP database. However, based on our observations, most readmissions were related to minor post-operative complications, such as UTI or SSI—as outlined in Table 3. Third, practical downstream consequences could not be assessed—such as potential economic strain for patients given the additional cost of mesh material, operating room time, and readmission. Fourth, although postoperative quality of life (QOL) is an important factor to consider, the ACS-NSQIP database does not currently include QOL metrics. Despite these limitations, this study's large size (in terms of patients, surgeons, and hospitals) provides a diverse sample that is more generalizable to the American population at large, relative to many other retrospective studies.

## 5 Conclusion

In this large retrospective cohort study, LHHR with mesh was associated with increased operative time, LOS, and hospital readmission at 30-days. However, there were no differences in mortality or overall morbidity. These findings provide much needed context to consider prior to employing mesh in LHHR. Large-scale randomized studies are needed to draw definitive conclusions regarding the safety profile and cost-benefit analysis of mesh use during LHHR.

## Data Availability

The data analyzed in this study is subject to the following licenses/restrictions: Access to the American College of Surgeons National Surgical Quality Improvement Program (ACS-NSQIP) patient registry dataset is restricted to individuals at participating hospitals who agree to the Data Use Agreement (DUA) and complete a request process. Requests to access these datasets should be directed to https://www.facs.org/quality-programs/data-and-registries/acs-nsqip/.
